# Noise Attenuation Estimation for Maximum Length Sequences in Deconvolution Process of Auditory Evoked Potentials

**DOI:** 10.1155/2017/3927486

**Published:** 2017-02-19

**Authors:** Xian Peng, Yun'er Chen, Tao Wang, Lei Ding, Xiaodan Tan

**Affiliations:** ^1^School of Biomedical Engineering, Southern Medical University, Guangzhou, Guangdong, China; ^2^Stephenson School of Biomedical Engineering, University of Oklahoma, Norman, OK, USA

## Abstract

The use of maximum length sequence (m-sequence) has been found beneficial for recovering both linear and nonlinear components at rapid stimulation. Since m-sequence is fully characterized by a primitive polynomial of different orders, the selection of polynomial order can be problematic in practice. Usually, the m-sequence is repetitively delivered in a looped fashion. Ensemble averaging is carried out as the first step and followed by the cross-correlation analysis to deconvolve linear/nonlinear responses. According to the classical noise reduction property based on additive noise model, theoretical equations have been derived in measuring noise attenuation ratios (NARs) after the averaging and correlation processes in the present study. A computer simulation experiment was conducted to test the derived equations, and a nonlinear deconvolution experiment was also conducted using order 7 and 9 m-sequences to address this issue with real data. Both theoretical and experimental results show that the NAR is essentially independent of the m-sequence order and is decided by the total length of valid data, as well as stimulation rate. The present study offers a guideline for m-sequence selections, which can be used to estimate required recording time and signal-to-noise ratio in designing m-sequence experiments.

## 1. Introduction

Maximum length sequence (m-sequence) has been found useful in the study of linear and nonlinear responsive components in the auditory system [1, 2]. Convoluted auditory evoked potentials (AEPs) can be elicited by an m-sequence of stimuli with its interstimulus intervals (ISIs) varying pseudorandomly. The cross-correlation technique has been developed to deconvolute linear/nonlinear components in AEPs [3, 4] from overlapped responses. The linear component reflects evoked responses to individual stimuli independently, and the nonlinear component reflects the temporal interaction of two or more stimuli. Therefore, the m-sequence method provides a unique tool in characterizing the human auditory system.

Usually, AEPs are highly contaminated with background electroencephalograms (EEGs) from various sources of noise or artifacts. An ensemble averaging technique has to be applied to enhance the signal-to-noise ratio (SNR) before deconvolution. It is well-known that noise power level is attenuated inversely proportional to number of signal sweeps to be averaged. The noise property of AEPs obtained using m-sequence can be studied from different perspectives. For example, Marsh [5] presented an intuitive explanation of noise constraints for m-sequence to extract the linear components of auditory brain stem response (ABR) using a subaveraging technique and demonstrated that ABR elicited by an m-sequence was noisier than conventional ABR obtained with same number of stimuli. Thornton [6, 7] presented a simple estimation method for SNR improvement using m-sequence in acquisition of otoacoustic emissions (OAEs). This estimation is based on an assumption of no adaptation effect for OAEs and estimated 3 dB SNR improvement for an m-sequence eliciting OAEs. Late on, Van Veen and Lasky [8] provided a general matrix-based framework for the response to arbitrary stimulus sequences and derived estimated SNR formula for m-sequences. Inspired by the success of m-sequence AEP, other deconvolution techniques have been rapidly advanced for various application scenarios (e.g., [9–11]). Delgado and Ozdamar [12] proposed a deconvolution method called continuous loop average deconvolution (CLAD), which provided a computational efficient solution to the problem and a capability of SNR estimation in the frequency domain. Based on the similar idea, they then employed Parseval's theorem to derive an SNR formulation for m-sequence and proved that m-sequence offers the highest SNR as compared with any CLAD sequences [13].

Conventionally, the raw EEG is epoched into EEG sweeps for averaging. A sweep of EEG is usually short in length equivalent to the ISI of the corresponding isochronic stimulus-sequence. The length of a signal sweep of m-sequence is much longer since EEGs to be averaged are in response to a full length of m-sequence containing a number of stimulus events, which is determined by the order of an m-sequence. The fact means that the number of EEG sweeps to be averaged has to be greatly reduced given a fixed EEG recording time, which gives rise to a problem of how to select the best m-sequence in terms of SNR. Although less number of sweeps will sacrifice SNR at the averaging step, the next cross-correlation step is expected to be able to attenuate more noise that may compensate its SNR loss. In the present study, we investigated the noise attenuation property of m-sequence with different orders using the cross-correlation technique. Based on the well-established noise attenuation relationship from the ensemble averaging process, we derived a noise attenuation ratio (NAR) metric for the m-sequence deconvolution procedure including both averaging and correlation processes. We then employed computer synthetic data and a real nonlinear AEP experiment to validate the proposed formula.

## 2. Method

### 2.1. Nonlinear m-Sequence Model

In general, a nonlinear system can be represented by a Volterra or Wiener series provided that the system is time-invariant with finite memory [14, 15]. The output of such a nonlinear system can be expressed by summations of multiorder convolutions of Volterra kernels:

(1) yt=h0+∫τ=0Th1τst−τdτ+∫τ1=0T∫τ2=0Th2τ1,τ2st−τ1st−τ2dτ1dτ2+⋯+∫τ1=0T⋯∫τp=0Thpτ1,…,τp·st−τ1⋯st−τpdτ1⋯dτp,

where *h*_1_(*τ*), *h*_2_(*τ*_1_, *τ*_2_),…, and *h*_*p*_(*τ*_1_,…, *τ*_*p*_) are the first, second, and *p*th-order Volterra kernels of the system; *T* is the system memory length; *s*(·) is the system input or the stimulation in this context. The Volterra kernels are equivalent to orthogonal Wiener kernels, which can be estimated by a method developed by Lee and Schetzen [16] using Gaussian white noise input. The Gaussian white noise input is unsuitable for transient AEPs that are usually elicited by individual short sound elements. Using binary m-sequence, Sutter [17] developed a computational efficient method to estimate the nonlinear kernels that are referred to as* binary kernels* based on the cross-correlation techniques [16]. Shi and Hecox [4] further extended it to m-pulse sequence which is in line with the linear application m-sequence firstly carried out by Eysholdt and Schreiner [3] in extracting the linear ABR at fast stimulus rate.

**Figure 1:**
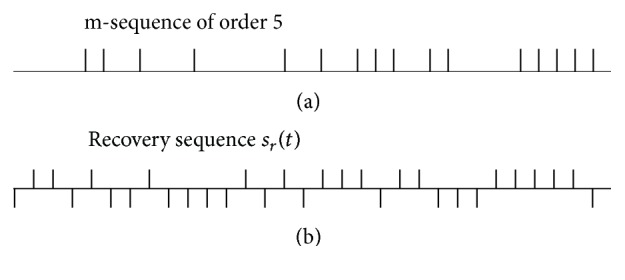
An instance of order 5 m-sequence (a) and the corresponding recovery sequence (b). The positive pulse train represents digit “1” of the m-sequence that indicates the occurrence of a sound stimulus, for example, a click. The negative pulse in the recovery sequence represents digit “−1” used to calculate the deconvolution process. The interpulse interval is padded with zeros to adjust the stimulation rate for specific application.

Mathematically, an m-sequence derived from a primitive polynomial is usually implemented by a number of shift-registers with different orders [18], say *r*. And the number of binary values or the length of an m-sequence is *L* = 2^*r*^ − 1. The m-pulse sequence proposed by Shi and Hecox [4] modified the m-sequence of binary element of {−1, +1} to a pulse sequence of {1,0} element, where the digit “1” is used to designate the occurrence of a transient stimulus, and digit “0” represents the silence of stimulation. In the discrete implementation of stimulations, the original m-sequence actually represents the most condensed stimulation rate that is practically unfeasible. Given the sampling rate *f*_*s*_ in practice, we have to sparsify an m-sequence by padding zeros between the neighboring binary elements. The number of zeros denoted by *q* is called sparse factor. In this case, the stimulation rates for an m-sequence can be derived from the reciprocal of the maximum ISI, the minimum ISI, and the mean ISI of an m-sequence. An instance of stimulation impulses derived from an order 5 m-sequence is shown in Figure 1. For every m-pulse sequence, a unique recovery sequence can be defined by an inverse operation on the original m-sequence (Figure 1). A unique mathematic property of m-sequence is that the cross-correlation function between the m-pulse sequence and the recovery sequence is an impulse function, which makes the deconvolution problem solvable and computational efficient.

According to the cross-correlation method modified by Shi and Hecox [4], all the impulse kernel slices are distributed within the cross-correlation signal *ϕ*(*t*) between the measured response *y*(*t*) to a sweep of m-sequence and the recovery sequence *s*_*r*_(*t*):

(2) $$\begin{array}{c} \phi \left(\;t\;\right)=\frac{2}{L+1}y\left(\;t\;\right)⊗s_{r}\left(\;-t\;\right). \end{array}$$

The onset location of the kernel slices is determined by a shifting function, which is determined* a priori* by the primitive polynomial used to generate the specific m-sequence. This method is essentially a deconvolution process for an inverse problem.

In practice, the m-sequence stimulation is delivered to human subjects repetitively, and the ensemble averaging is applied to epoched responses to enhance SNR before the cross-correlation analysis. Thus, the NAR in dB is

(3) $$\begin{array}{c} \eta_{a}=20\;\log_{10}\left(\;\frac{\sigma_{a}}{\sigma_{n}}\;\right)=-10\;\log_{10}\left(\;K\;\right)\left(\;dB\;\right), \end{array}$$

where *K* is the number of EEG sweeps to be averaged. *σ*_*a*_ and *σ*_*n*_ are the root mean square (RMS) values of averaged and raw EEG data, respectively. Suppose the same EEG recording time; the *K* will be different for m-sequences of different order *r*:

(4) $$\begin{array}{c} K=\frac{N}{Lq}=\frac{N}{\left(2^{r}-1\right)q}, \end{array}$$

where *N* is the length of raw EEG signal in response to a number of m-sequence stimulations, and *q* is the sparse factor used to adjust ISI in discrete time implementation. It is noted that lower order m-sequences attenuate noise much better. Nevertheless, such a benefit might be neutralized in the next correlation analysis step. Considering that measured response *y*(*t*) to a sweep of m-sequence contains additive noise, that is, *n*(*t*), which is unrelated to stimulus events, and that *s*_*r*_(*t*) is a train of *L* pulse functions (positive and negative pulses, see Figure 1), the convolution operator in (2) is essentially a superposition of *y*(*t*) with its moving version of different signs. Suppose that *σ* denotes the RMS value of a signal before the correlation analysis; the noise term of *y*(*t*) ⊗ *s*_*r*_(−*t*) will be $\sqrt{L}\sigma$ therefore, since it is equivalent to a moving summation. Thus, combining the coefficient term 2/(*L* + 1) in (2), the NAR after (2) should be

(5) ηϕ20 log10⁡σϕσa=20 log10⁡2LL+1≈20 log10⁡2−10 log10⁡LdB.

The approximation holds for *L* ≫ 1. The overall NAR is thus given by

(6) ηϕaηϕ+ηa=20 log10⁡2LqL+1N≈C−10 log10⁡NdB,

where *C* = 20log⁡2 + 10log⁡*q* can be considered as a constant given a fixed *q* in an application. This simple equation indicates that the overall noise attenuation is only determined by the length of recording signal. The m-sequence order does not affect such a noise property. The sparse factor *q* controls the average stimulation rate, which is an important parameter in applications. An arbitrary stimulation rate can be achieved by adjusting both *q* and the sampling rate.

### 2.2. Simulation Experiments

Since the magnitude of genuine responses may vary with respect to the stimulation property, for example, due to the adaptation effect of nervous systems, the SNR property was not investigated using a specific experiment. Instead, noise attenuation property was concerned by calculating the ratio of RMS amplitude for a target noise. To examine noise attenuation through the average and cross-correlation processes, background EEGs were simulated with various pink noise of 1/*f* power distribution, which was considered as an appropriate model for EEG characteristics [19]. In the present study, EEGs corresponding to m-sequences of order *r* = {5,6,…, 12}, sparse factor *q* = 40 at 20 kHz sampling rate, equivalent to 2 ms minimum ISI, were synthesized. Simulated EEGs of around 14 min in length were chosen. The exact length varied a bit to guarantee integral multiple sweeps that was the length of an m-sequence. This length corresponded to 100 sweeps for the m-sequence of order 12. Since EEGs were generated randomly, each NAR presented as a mean value over 15 EEG samples, and the standard deviations (sd) were also presented in necessary.

### 2.3. Real Nonlinear Experiments

Real EEGs were acquired from human subjects stimulated by m-sequences of order 7 and 9 (same sequences used in the previous simulation experiment). Adult subjects were recruited and given informed consent approved by the Institutional Review Board of Southern Medical University. Nine subjects (age 21–23, six males) were enrolled with normal hearing. EEG data were acquired using SynAmps^2^ amplifier (Compumedics Ltd., Victoria, Australia) at the sampling rate of 20 kHz and a 100–2000 Hz (12 dB/oct) bandpass filtering. Three Ag/AgCl surface electrodes were placed on the upper forehead (active), lower forehead (ground), and ipsilateral mastoid (reference) with electrode impedances of less than 5 kΩ. Subjects were seated on a deckchair in an electromagnetic shielded and soundproof booth during EEG recording. Rarefaction clicks were delivered monaurally to the right ear at 82 dB pSPL via an insert earphone (ER-3A Etymotic Research, Elk Grove Village, IL, USA).

EEGs were recorded continuously in response to stimuli sweeps of an m-sequence and repeated for two runs. Each run contained 2000 sweeps for order 7 m-sequence and 500 sweeps for order 9 m-sequence. The length of EEG per run was about 8.47 min for order 7 sequence and 8.52 min for order 9 sequence.

## 3. Results

### 3.1. Simulation Results

**Figure 2:**
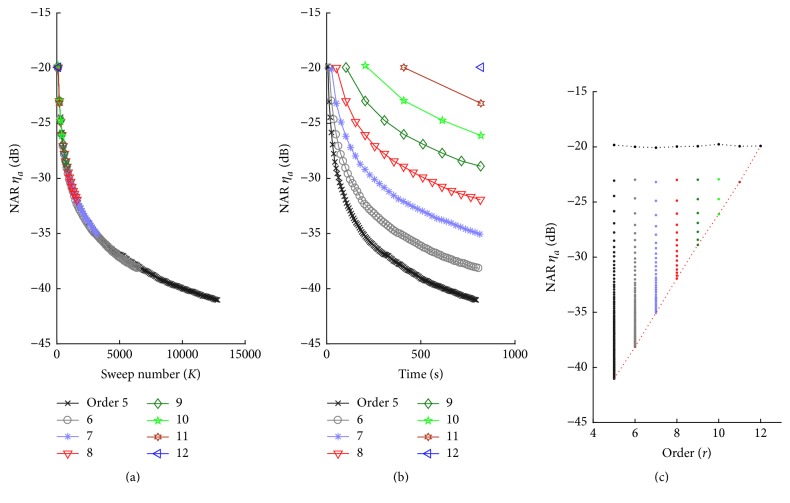
NARs of the average process with respect to sweep number (a), EEG recoding length (b), and the m-sequence orders (c) for the simulated EEGs corresponding to 5–12 order m-sequences. The total recoding lengths of these EEGs are about the same, which results in different sweep numbers to be averaged.

The noise attenuation in the averaging process is determined by the number of sweeps to be averaged. In this experiment, the averaging number is dependent on the m-sequence order when the length of EEG recordings was same for different sequences.  Figure 2(a) shows the NARs with respect to the number of averaging sweeps, which demonstrates its inversely proportional pattern to the square root of the sweep number *K* as indicated in (3).  Figure 2(b) shows these NARs rescaled with respect to the time or sweep length for different order m-sequences, which demonstrates that more sweep numbers available for lower order m-sequence lead to different levels of NAR given same EEG recording lengths. As the EEG sweep length varies, which is equivalent to the time incremental spaces in these traces (Figure 2(b)), Figure 2(c) shows these ratios with respect to the m-sequence orders from 5 to 12. The lower border line (the red dotted line) indicates the attenuation ratios averaged over the same entire recording time (about 14 min in these cases), and the upper border line (the black dotted line) indicates the least number of averaging sweeps (100 in these cases). These figures present the NAR properties for the averaging process in various perspectives, which coincide well with the theoretical ones as defined in (3).

**Figure 3:**
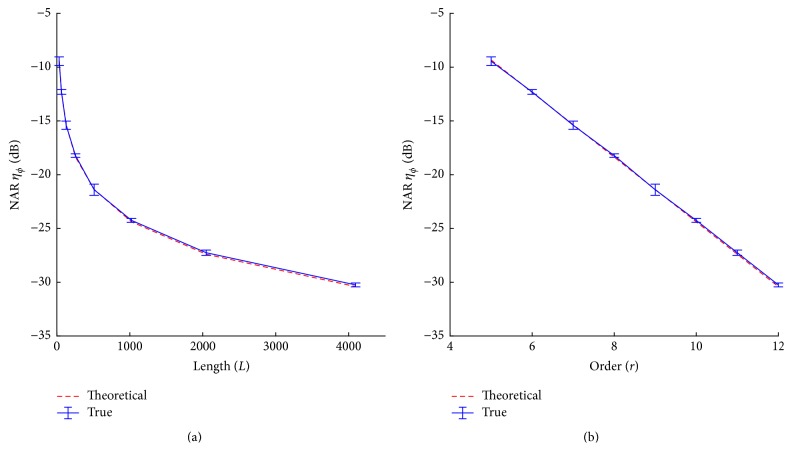
NARs (*m* ± sd) of the correlation process for m-sequences of orders 5–12.

As indicated in (5), the cross-correlation process attenuates noise as well, as determined by the element number of m-sequence, *L*, which means that the higher order m-sequences with larger *L* lead to more noise attenuation. Simulated NARs (*m* ± sd) of this process are shown in Figure 3 for the same data set as shown in Figure 2. The inversely proportional relationship between *L* and NAR can be observed in Figure 3(a). *L* exponentially increases with the m-sequence order, since *L* = 2^*r*^ − 1. A linear increased m-sequence order *r* for these data is shown in Figure 3(b), which illustrates a linear relationship of NAR to the m-sequence order. Figure 3 also shows that the simulated results coincide with the theoretical ones very well (red dashed lines).

**Figure 4:**
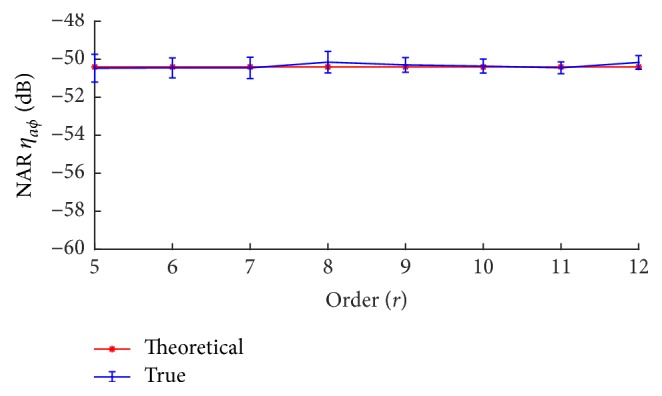
NARs (*m* ± sd) of the whole processes of averaging and correlation for the same EEG length of about 14 min.

Since the averaging and correlation processes attenuate noise differently with respect to the sequence order, the effect of both processes can be observed under the condition with same total recording times for different m-sequence orders. As indicated in (6), the ratio is only determined by the total length of EEG signal involved in the deconvolved computation including both averaging and correlation processes.  Figure 4 shows the simulated and theoretical NARs of the whole deconvolution process for EEG recordings of 14 min. Both the theoretical analysis and simulation results prove that the m-sequence order will not affect the SNR in the deconvolution process given the same recording time. The averaging and correlation balance noise attenuation effect to a rational level that is only dependent on data recording times, but not the sequence order.

### 3.2. Real AEPs for 7 and 9 Order m-Sequences

**Figure 5:**
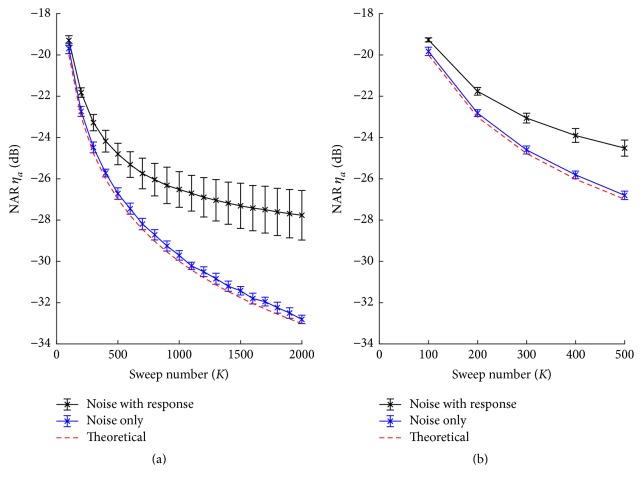
NARs of the averaging process for order 7 (a) and order 9 (b) m-sequences using the conventional averaging process that contains evoked components and the alternative reference averaging process.

The important difference between a real AEP experiment and simulation experiment is that acquired EEGs in the real AEP experiment contain both background noise and stimulus evoked components. The averaging and correlation processes cannot attenuate evoked components that might cause errors in calculating NARs. A simple way to estimate pure noise term is to use the alternative reference technique [20], which adds and/or subtracts EEG sweeps alternatively to cancel out phase-locked responses.  Figure 5 shows the mean NARs with standard deviation (*m* ± sd) from 9 subjects with respect to the number of sweeps in the averaging process. Conventional averaging process using EEG containing responses (black lines) shows much less NARs and larger deviations from the theoretical NARs (red dashed lines). However, the NARs calculated after the alternative reference method for noise only estimation (blue lines) show much close patterns to the theoretical ones (red dashed lines). The maximal sweep numbers of about 8.5 min raw EEGs used for order 7 and 9 cases are 2000 and 500, respectively.

**Figure 6:**
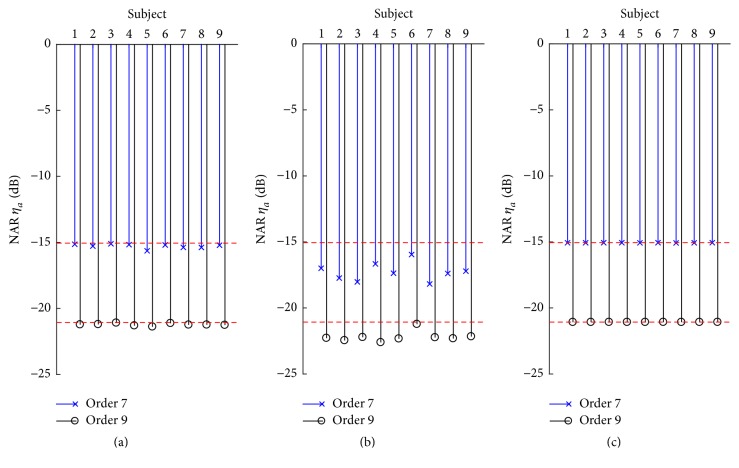
NARs of correlation for order 7 (blue “—×” lines) and 9 (black “—○” lines) m-sequences for 9 subjects and under three calculation conditions: responses involved by conventional averaging and correlation processes (a), excluding responses from correlation process (b), and excluding responses from both correlation and averaging processes.

Unlike averaging process, the correlation process can circumscribe the onset locations of evoked responses for both linear and nonlinear components. A location within the correlation signal can be given by a shifting function to locate the onset site of a component [4]. A memory length for these responses can then be defined to isolate them, which are usually termed kernel slices, since they represent a response along the diagonal dimensions. Therefore, evoked responses can be excluded from correlation signals using such a method. The NAR for the correlation process can be calculated at three conditions as shown in Figure 6, where NAR values of each subject are indicated by a bar plot. The theoretical NARs are about −21 dB for order 7 sequences and −15 dB for order 9 sequences (red dashed lines).  Figure 6(a) presents the NARs in the correlation analysis based on the condition of the conventional averaging process over the maximum sweep number, which means that evoked responses are not rejected in both averaging and correlation analyses. However, the NARs are quite well consistent with the theoretical ones for all subjects, particularly in comparison with Figure 6(b), where evoked responses are excluded from the correlation analysis using shifting functions, but not in the averaging analysis. These results can be attributed to the fact that evoked responses remain in the averaging step, which affect *σ*_*ϕ*_ and *σ*_*a*_ as in (5). It suggests an interesting fact on NARs that it can be approximately estimated with acceptable accuracy from EEG data obtained in real AEP experiments without considering the effect of evoked responses. Nevertheless, a more accurate estimation can be achieved by excluding evoked responses in both the averaging and correlation processes (Figure 6(c)).

**Figure 7:**
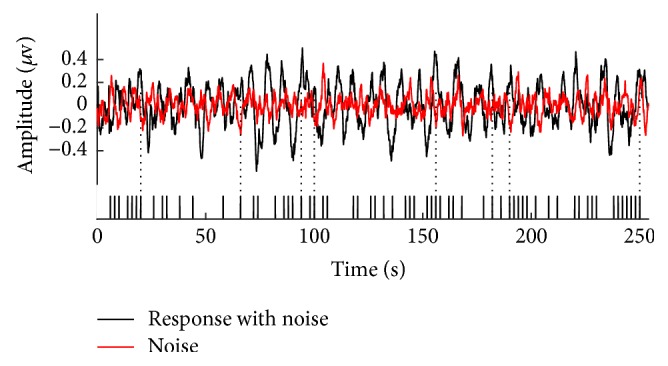
A typical instance of the averaged response for a subject by conventional averaging (black) and noise only averaging by alternative reference method (red). The m-sequence on the bottom indicates some correspondences between the stimulus clicks and the distinct response that may be identified.

To illustrate the evident responses after averaging, we present an instance of averaged signals of order 7 m-sequence for subject 3 in Figure 7. The averaged response (back trace) shows distinct larger amplitude than the noise signal estimated by the alternative reference method (red trace)—about 6 dB difference specifically in this case. It is also possible to identify some distinct responses corresponding to onsets of stimulus clicks in the m-sequence indicated by the dotted lines. These responses may contribute mainly to the linear AEP components.

**Figure 8:**
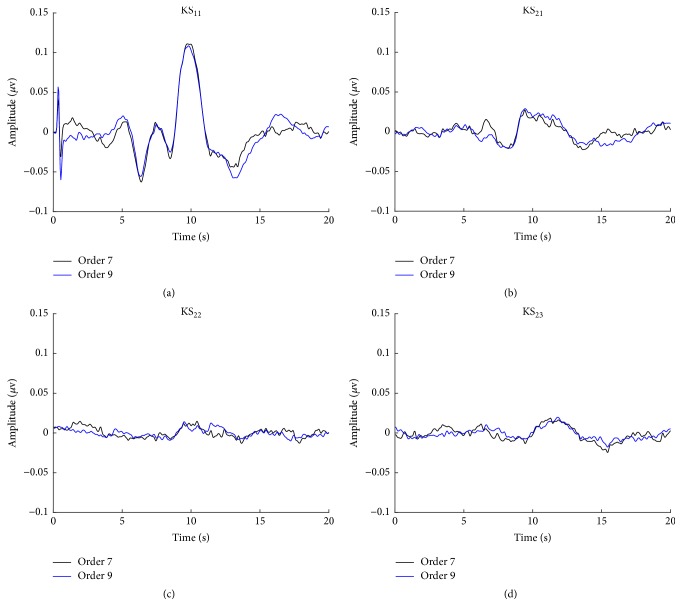
Linear and nonlinear AEP components extracted by two m-sequences, where KS_11_ denotes the response of linear kernel slice, and KS_21_–KS_23_ represent three second-order nonlinear responses.

Figure 8 illustrates the extracted linear and nonlinear components averaged over all subjects for two m-sequences. The figure shows almost identical linear AEPs or the main diagonal kernel slice (KS_11_ in Figure 8(a)) and three well-matched second-order nonlinear AEPs (KS_21_–KS_23_ in Figures 8(b)–8(d)). The linear component reflects the neural responses to individual clicks that consist of the largest energy in responses, whereas the second-order nonlinear components reflect the temporal interaction between neighboring clicks that are only about one-tenth amplitude as compared with the linear component, indicating being potentially more susceptible to noise contamination. These results demonstrate an agreement in terms of SNR for m-sequences of different orders.

## 4. Discussion

In the application of using m-sequence to investigate both linear and nonlinear evoked components, it is faced with a problem of selecting m-sequence with different orders and different mathematical properties (or primitive polynomial). It is essential to know these differences and their influences on experimental results. The present study reports an effort of assessing m-sequences on their noise attenuation property. A simple formula is derived to estimate noise attenuation ratio for a typical way of delivering m-sequence stimulations based on the well-established relationship of averaging theory on additive noise conditions.

Previous studies claimed that m-sequence may reduce recording time since rapid stimulation is achieved in the linear deconvolution process with m-sequence [3, 21]. This effect can also be explained and estimated by (6), which is equivalent to reduce sparse factor *q* in order to suppress the noise term. As an example, if the mean ISI of an m-sequence is 2*q*, this means that the noise is $\sqrt{2}$ times larger than the equivalent isochronic stimulation with the same ISI. However, SNR may be balanced by suppressed response due to the adaptation effect of nervous systems at rapid stimulation rates [22]. Since different AEP components may exhibit various adaptation effects, some components may even be enhanced if they are sensitive to the jitter in m-sequence [23, 24]. It is therefore not wise to investigate SNR under such conditions.

Although the present study reports that the order of an m-sequence will not affect noise attenuation provided that entire EEG recording times are same, the selection of m-sequence of the same order and the selection of m-sequence order are also very important in practice. As is known, the mathematic property of m-sequence is totally dependent on the primitive polynomial used to generate the sequence. There are a fixed number of primitive polynomials for an order. A previous study reported that kernel slices distribute differently on correlation signals that may cause distortions for some slices from overlapped responses [25]. Higher order m-sequences with long correlation signals naturally have a larger tolerance on more kernel slices. However, there are also practical concerns in using long m-sequences. As an example, when dealing with artifacts, entire EEG sweeps might have to be rejected, which leads to disadvantage in using long m-sequences.
